# A telemedicine-enabled intravenous thrombolytic treatment pathway for patients with hyperacute non-arteritic central retinal artery occlusion

**DOI:** 10.1016/j.ajoc.2024.102204

**Published:** 2024-10-20

**Authors:** Aubrey L. Gilbert, Amar P. Patel, Dana Sax, M. Tariq Bhatti, Ronak Shah, Adrian Dokey, Tova Mannis, Molly Burnett, Robin A. Vora

**Affiliations:** a Kaiser Permanente Northern California, Department of Ophthalmology, Vallejo, CA, USA; b Kaiser Permanente Northern California, Department of Ophthalmology, Oakland, CA, USA; c Kaiser Permanente Northern California, Department of Emergency Medicine, Oakland, CA, USA; d Kaiser Permanente Northern California, Department of Ophthalmology, Roseville, CA, USA; e Renaissance School of Medicine at Stony Brook University, Stony Brook, NY, USA; f Kaiser Permanente Northern California, Department of Neurology, Oakland, CA, USA

**Keywords:** Central retinal artery occlusion, Retinal vascular disease, Embolic disease, Stroke, Intravenous thrombolysis, Tenecteplase, Telemedicine

## Abstract

**Purpose:**

To describe the visual acuity (VA) outcomes from a telemedicine-enabled pathway allowing for rapid diagnosis and administration of intravenous (IV) thrombolytic treatment for non-arteritic central retinal artery occlusion (naCRAO) within 4.5 hours (4.5 h) of visual loss.

**Design:**

Retrospective observational case series.

**Methods:**

Setting: A large managed healthcare consortium.

Patient Population: Eighty-five patients with naCRAO and vision loss for less than 4.5 h presenting between 2021 and 2023. Thirty-five patients received IV thrombolytic therapy and 50 patients were closely observed.

Intervention: A collaborative telemedicine-enabled pathway employing fundus photography was previously established by Ophthalmology, Emergency Medicine, and Stroke services to rapidly evaluate and manage patients presenting with acute painless monocular vision loss and allow for administration of IV tenecteplase (0.25 mg/kg) for eligible consenting patients diagnosed with naCRAO within 4.5 h. Retrospective chart review was conducted to collect data on demographics, vascular risk factors, clinical features, VA outcomes, and adverse events. Comparison was made between patients who received intravenous thrombolysis and those who were observed.

Main Outcome Measures: Improvement in VA of at least 0.3 logarithm of the minimum angle of resolution (logMAR) and/or from 20/200 or worse to 20/100 or better.

**Results:**

A greater percentage of patients in the treated group had VA improvement of ≥0.3 logMAR (54.3 % vs 28 %, p = .014), and a greater percentage of patients in the untreated group had VA worsening of ≥0.3 logMAR (30 % vs 5.7 %, p = .006). Twice the percentage of treated versus untreated patients had improved VA from 20/200 or worse to 20/100 or better, but this difference was not statistically significant (20 % vs 10 %, p = .192). There was a significantly shorter mean time to treatment for those patients who had VA improvement from 20/200 or worse to 20/100 or better compared to those who did not (118 versus 171 min, p = .031). Two patients experienced intracranial bleeding after IV thrombolysis.

**Conclusions:**

The evaluation and treatment of hyperacute naCRAO is possible on a large scale via an integrated telemedicine-enabled approach utilizing fundus photography. The use of IV thrombolytic was associated with better VA outcomes compared to observation alone. Prospective randomized controlled trials are needed to confirm these findings and determine optimal management.

## 1 Introduction

Non-arteritic central retinal artery occlusion (naCRAO) is a central nervous system stroke that generally results in severe monocular visual loss.^1^^,^^2^ A recent study examining the natural history of naCRAO showed that less than 8 % of patients with visual acuity (VA) of 20/200 or worse on presentation improve to 20/100 or better. Furthermore, hyperacute presentation, in less than 4.5 h after symptom onset, is not associated with better VA outcomes, nor is the use of conservative interventions such as anterior chamber paracentesis, ocular massage, intraocular pressure (IOP) lowering drugs, or hyperventilation.^2^

Intravenous (IV) thrombolysis within 4.5 h of symptom onset is the current standard of care for acute ischemic cerebral stroke.^1^^,^^3^ Given common underlying pathophysiologic mechanisms, it stands to reason that the same treatment might have efficacy for naCRAO.^4^^,^^5^ To date, there have been no results from large-scale randomized controlled trials investigating IV thrombolysis for hyperacute naCRAO, but meta-analyses of observational studies have suggested a significantly higher rate of VA recovery in patients receiving IV thrombolysis within 4.5 h of vision loss compared to the natural history of the disease.^6^, ^7^, ^8^ Although not level 1 data, such evidence recently prompted the Centers for Medicare and Medicaid Services (CMS) to create a new diagnosis related group for CRAO patients treated with thrombolysis.^9^

While several randomized controlled trials are underway in Europe,^10^ recruitment has been hampered due to the low incidence of naCRAO and the infrequency of diagnosis within the potential therapeutic window. Our group determined that up to 51 % of acute naCRAO patients in our integrated healthcare system made contact within 4.5 h after vision loss. However, time to ophthalmic consultation and diagnosis lagged far behind.^11^ To facilitate more expedient ophthalmologic diagnosis, a collaborative effort was undertaken by our Emergency Medicine, Ophthalmology, and Stroke services. Herein we describe our recently developed telemedicine-enabled algorithm, employing remote evaluation of fundus photographs and telestroke care, and report VA outcomes for patients with naCRAO presenting within 4.5 h of visual loss who received IV thrombolytic therapy in comparison to a similar cohort of patients who did not receive IV thrombolytic therapy.

## 2 Methods

**Fig. 1:**
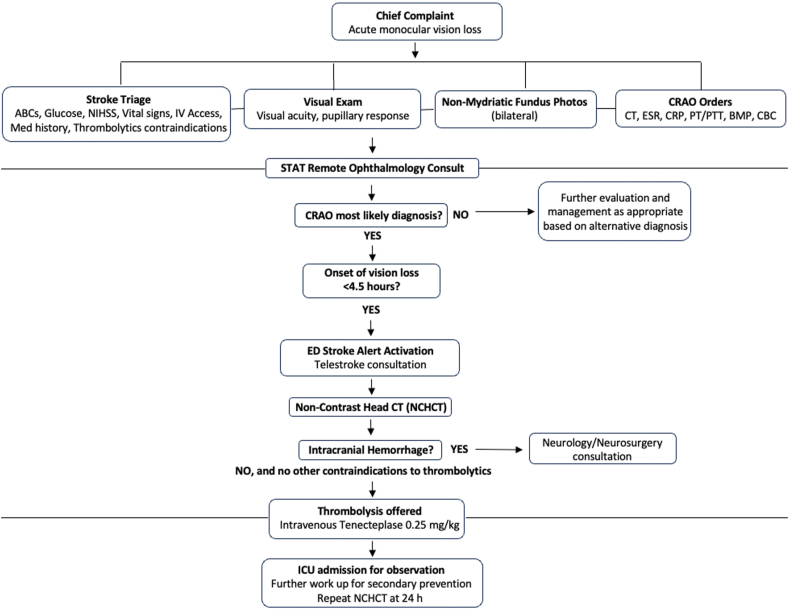
Management pathway for patients presenting within 4.5 h of acute painless monocular vision loss. Thrombolysis was only offered for visual acuity of 20/200 or worse.

Kaiser Permanente Northern California (KPNC) is an integrated healthcare and coverage program serving over 4.5 million people who represent state and national racial and socioeconomic diversity.^12^^,^^13^ It includes 21 hospitals and 207 medical offices and outpatient facilities across the northern and central regions of the state. Beginning in early 2022, nonmydriatic fundus cameras (iCare DRSplus, Icare USA, Raleigh, NC) were consecutively introduced into 21 KPNC emergency departments (EDs). Each camera was connected to the ophthalmic enterprise imaging system, (Sectra Ophthalmology, Sectra, Linköping, Sweden) which enables remote image viewing to allow for immediate evaluation by offsite ophthalmologists. A team of doctors from multiple specialties collaborated to develop a management pathway utilizing these cameras for patients with acute monocular vision loss (Fig. 1).

ED triage teams were instructed to treat monocular vision loss as a sign of potential stroke, activating stroke code protocols which permit for rapid and parallel testing. Upon initial presentation, patients underwent stroke triage, VA measurement, and non-mydriatic fundus photography. The on-call comprehensive ophthalmologist was consulted to discuss the clinical history and review fundus images remotely, on either their KPNC-issued mobile device or computer. If naCRAO was deemed the most likely diagnosis, the telestroke team^14^ was contacted and an urgent computed tomography (CT) of the head was performed to identify any findings that might present increased risk for intracranial hemorrhage in the context of thrombolytic administration. Finally, if the patient remained within the pre-defined therapeutic window of 4.5 h, had measured VA of 20/200 or worse, and met all other IV thrombolytic candidacy criteria, thrombolysis was offered by the telestroke team. If patient consent for this experimental therapy was granted, an IV bolus of tenecteplase at a dose of 0.25 mg/kg was administered. After treatment, patients were admitted to the intensive care unit for monitoring and evaluation for modifiable risk factors for secondary prevention.

In early 2024, the electronic medical record (Epic Systems Corporation, Verona, WI) was queried using International Statistical Classification of Diseases and Related Health Problems, Tenth Revision codes (ICD-10-CM)^15^ to identify all patients newly diagnosed with CRAO between January 1st, 2021 and December 31st, 2023, a period including introduction of the expedited pathway described above. Two retina specialists (RAV and APP) reviewed all clinical notes and retinal imaging studies to confirm the diagnosis of naCRAO. Both specialists needed to agree for study inclusion. If consensus was not possible, the patient was excluded. Cases with any question regarding a diagnosis of arteritic CRAO, based on clinical history, laboratory testing, or other studies, were also excluded. A retrospective chart review was performed to gather data on demographics, vascular risk factors, details of presentation, evaluation, and management, measured VAs, and any adverse events. Focus was placed on those naCRAO patients who presented within 4.5 h of vision loss. Data was additionally gathered from both the initial visit in the Ophthalmology clinic after CRAO as well as the follow-up visit closest to 90 days after vision loss. Although the study window was 2021–2023, the treatment pathway was not fully enacted until mid-2022. The majority of untreated patients in the study presented prior to the time when treatment was being offered. Of those who presented after the time when treatment was available, almost all who did not receive it were ineligible based on various contraindications determined by the evaluating stroke neurologists.

Categorical variables were evaluated by chi-squared tests, while continuous variables were evaluated by t-tests, and a linear regression analysis was performed to evaluate change in visual acuity by time to treatment. Snellen visual acuities were converted to logarithm of the minimum angle of resolution (logMAR) to facilitate data analysis, with no light perception as 3 logMAR, light perception as 2.7 logMAR, hand motions as 2.28 logMAR, and counting fingers as 1.85 logMAR.^16^^,^^17^ Improvement of VA was evaluated in terms of a decrease of at least 0.3 logMAR and/or a transition from 20/200 or worse to 20/100 or better. Worsening of VA was defined as an increase of at least 0.3 logMAR. A p value of <0.05 was considered significant. This study was approved by the KPNC Institutional Review Board and adhered to the Declaration of Helsinki. Informed consent was not obtained for this study as deidentified data were used.

## 3 Results

Among all patients assigned an ICD code of CRAO between 2021 and 2023, 295 were confirmed to have an accurate diagnosis of naCRAO presenting within 30 days of vision loss. Two hundred and five patients presented within 24 h of vision loss, 131 patients presented within 12 h, 95 patients presented within 6 h, and 86 patients presented within 4.5 h. For these 86 patients, the mean age at time of presentation was 72 years (range 39–97 years) 60.5 % were male, and 46.5 % were affected in the right eye. Thirty-five of the 86 patients received IV tenecteplase. The treated group of patients did not differ significantly from the untreated patients in terms of age, sex, race, or vascular risk factors (Table 1). Since one patient in the untreated group was lost to follow-up after presentation, VA analysis was conducted on 85 patients. There was one additional patient who received IV tenecteplase who was later determined to have arteritic CRAO. This patient was not included in VA analysis, but they experienced neither any improvement in visual acuity nor any adverse events.

**Table 1:** Patient characteristics.

Characteristic	All patients (n = 86)	No treatment (n = 50)	Intravenous Thrombolysis (n = 35)	p-value
Sex				
Male	52 (60.5 %)	31 (60.1 %)	21 (60 %)	0.5
Age at presentation				
Mean (range)	72 (39–97)	71 (39–97)	74 (41–93)	0.79
Race				
Non-white	23 (26.7 %)	12 (23.5 %)	11 (31.4)	0.4
Laterality				
Right eye	40 (46.5 %)	22 (43.1 %)	18 (51.4)	0.5
Co-morbid illnesses				
Hypertension	68 (79.1 %)	39 (76.5 %)	29 (82.9 %)	0.59
Hyperlipidemia	55 (64.0 %)	31 (60.8 %)	24 (68.6 %)	0.89
Diabetes Mellitus	30 (34.9 %)	17 (33.3 %)	13 (37.1 %)	0.9
Coronary artery disease	20 (23.3 %)	14 (27.5 %)	6 (17.1 %)	0.32
Atrial fibrillation	16 (18.6 %)	10 (19.6 %)	6 (17.1 %)	0.9
History of smoking	53 (61.6 %)	33 (64.7 %)	20 (57.1 %)	0.5

The mean presenting VA was worse in the treated group than in the untreated group (2.40 logMAR versus 2.05 logMAR, p = .005). A greater percentage of patients in the treated group had improvement of VA by at least 0.3 logMAR (54.3 % versus 28 %, p = .001), and a greater percentage of patients in the untreated group had worsening of VA by at least 0.3 logMAR (30 % versus 5.7 %, p = .006). Compared to the untreated group, twice the percentage of patients in the treated group improved from VA of 20/200 or worse to 20/100 or better, (10 % versus 20 %) but this difference did not achieve statistical significance (p = .193). There was a significant difference in change in VA from presentation to follow up between the untreated and treated patients, with no significant change in VA for patients in the untreated group, and a mean improvement of −0.589 logMAR for patients who received IV thrombolysis (p = .002) (Table 2).

**Table 2:** Visual acuities for the treated and untreated patient groups.

	No treatment (n = 50)	Intravenous thrombolysis (n = 35)	p-value
Presenting VA (mean logMAR)	2.05	2.40	0.005∗
Follow up VA (mean logMAR)	2.01	1.81	0.227
Change in VA (mean logMAR)	0.04	−0.59	0.002∗
Improved by ≥ 0.3 logMAR	14 (28 %)	19 (54.3 %)	0.014∗
Improved from 20/200 or worse to 20/100 or better	5 (10 %)	7 (20 %)	0.193
Worsened by ≥ 0.3 logMAR	15 (30 %)	2 (5.7 %)	0.006∗

VA = visual acuity.

logMAR = logarithm of the minimum angle of resolution.

∗p < .05.

**Fig. 2:**
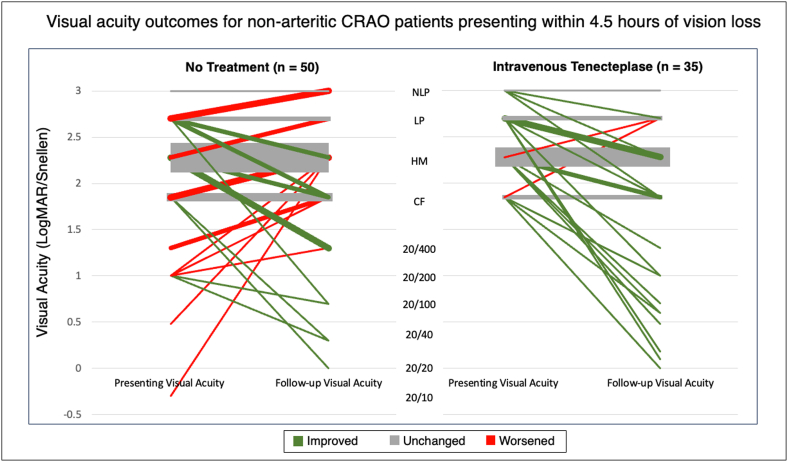
Initial and follow up visual acuities for non-arteritic central retinal artery occlusion patients presenting within 4.5 h of vision loss. Line thickness is scaled to number of patients for a given visual acuity. Lines are color coded by direction of change in visual acuity for each patient, with patients whose visual acuity (VA) improved in green, those whose VA remained the same in grey, and those whose VA worsened in red. Many patients had no change in measured VA. More patients had improved VA and fewer patients had worsened VA in the treated group compared to the untreated group. CRAO = central retinal artery occlusion; LogMAR = logarithm of the minimum angle of resolution. (For interpretation of the references to color in this figure legend, the reader is referred to the Web version of this article.)

In the treatment group, there was no significant difference in the mean follow up logMar VA at initial follow up (on average around 2 weeks after treatment) compared to follow up closer to 3 months after treatment (1.83 logMAR versus 1.77 logMAR, respectively, p = .774). As all of the treated patients presented with VA of counting fingers or worse, a subgroup analysis including only the 41 untreated patients who presented with VA of counting fingers or worse was performed. There was no significant difference in mean presenting VA between this subgroup and the treated group (2.31 logMAR versus 2.40 logMAR, p = .239). Comparison of this subgroup with the treated group otherwise yielded similar results to comparison of the treated group with the full cohort of 50 untreated patients, although there was a less significant difference in percentage of patients with worsened VA. While 5.7 % of the treated patients worsened by at least 0.3 logMAR, 29.4 % of the entire untreated cohort (p = .006), and 19.5 % of the subgroup of 41 untreated patients (p = .076) worsened by at least 0.3 logMAR. VA measurements for all 85 patients are displayed in Fig. 2.

**Fig. 3:**
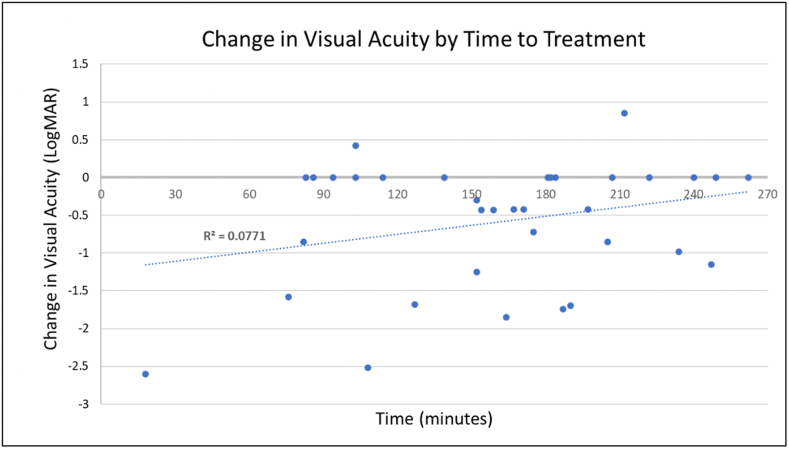
Change in visual acuity by time to treatment. There was a non-significant trend in change in visual acuity (VA) by time to treatment, with a shorter time to treatment associated with greater improvement in VA. LogMAR = logarithm of the minimum angle of resolution.

For the treatment group, the average time from vision loss to tenecteplase treatment was 159 min, with a median time to treatment of 154 min and a range from 18 to 262 min. In the case of the patient who received tenecteplase 18 min after vision loss, the diagnosis of CRAO was presumptive and made without fundus photography, as he was already in the ED being evaluated for acute embolic cerebral stroke at the time of vision loss. The treated patients who had at least 0.3 logMAR improvement in VA had a shorter mean time from vision loss to treatment than the treated patients who did not, but this difference did not achieve statistical significance (157 min versus 166 min, p = .653). However, there was a significantly shorter mean time to treatment for those treated patients who had improvement in VA from 20/200 or worse to 20/100 or better compared to those who did not (118 min versus 171 min, p = .031). There was a non-significant trend of change in VA by time to treatment (Fig. 3).

Fundus photography was diagnostic of CRAO in 84 % of images obtained. Of the 16 % of images deemed non-diagnostic, half were uninterpretable (blurry images and/or uncaptured central fundus). The other images were interpretable, but not clearly diagnostic of CRAO.^18^ The earliest a fundus photograph was obtained after vision loss was 44 min. There was a significantly shorter time from vision loss to photography for the interpretable but non-diagnostic photographs compared to the ones that were diagnostic (64 min versus 133 min, p = .001), consistent with the fact that it takes some time for clinically evident retinal edema to become apparent following CRAO. This may correlate with there being less ischemia at the time of photography for the cases that were caught sooner.

Two patients in the treatment group experienced intracranial bleeding despite appropriate pre-screening for any bleeding diatheses and the absence of any evident cerebral ischemia on pre-treatment head CT. This imaging was reviewed again and, even in retrospect, there was no apparent ischemia, but it is likely that they had small foci undetectable by CT. Both were managed conservatively with serial imaging. One patient had a cerebellar hematoma with adjacent subarachnoid hemorrhage that resolved without sequelae, and the other had occipital bleeding resulting in a persistent superior quadrantanopia.

## 4 Discussion

The natural history of naCRAO carries a poor visual prognosis.^1^^,^^2^ While IV thrombolysis has shown promise for improved visual outcomes in retrospective case series and patient-level meta-analyses,^6^, ^7^, ^8^ there remains a lack of data from large randomized controlled trials and as such it is not included in the AAO preferred practice guidelines. Still, there is felt to be clinical equipoise to offer it,^5^ but the need to evaluate patients for treatment within a short period of time after vision loss presents a major hurdle. We have successfully implemented an integrated telemedicine-enabled pathway leveraging fundus photography to expedite evaluation and management of these patients on a large scale, and we detail the more favorable visual outcomes observed in a cohort receiving experimental IV thrombolysis through this pathway compared to an untreated cohort.

While untreated patients demonstrated no significant change in mean VA from presentation to follow up, patients who received IV thrombolysis had an average VA improvement of −0.589 logMAR. While a lower percentage of the treated patients in our study had worsened VA at follow up compared to the untreated patients, the treated patients did have a worse mean presenting VA compared to the untreated group, so a floor effect may account for some of that discrepancy. However, VA outcomes were still better in the treated group even in comparison to the subgroup of 41 untreated patients who started with a more similar average VA to the treated group.

The percentage of treated patients with VA improving by at least 0.3 logMAR or from 20/200 or worse to 20/100 or better was around twice that of patients in the untreated group. These percentages are not as high as those reported in recent meta-analyses for other pooled naCRAO cohorts treated with IV thrombolysis. 54.3 % of treated patients in our study improved by at least 0.3 logMAR, versus 74.3 % in a recent large meta-analysis,^8^ and 20 % of treated patients in our study improved from 20/200 or worse to 20/100 or better, versus 39 % in a recent large meta-analysis.^8^ It is possible that this difference is due to our treated cohort having worse mean presenting VA than the patients in some of the studies included in the meta-analysis, although better outcomes have been reported even for cohorts with similar mean presenting VA and treatment time to ours.^7^^,^^19^ The reason for this is unclear but may be a consequence of relatively small sample size.

Regarding adverse events, of the 35 patients in our treated cohort, 2 (5.7 %) experienced intracranial bleeding post-thrombolysis. This is within an expected range based on published data regarding bleeding risk with IV thrombolysis for acute ischemic stroke.^20^, ^21^, ^22^ One patient was left with a permanent deficit, that of a superior quadrantanopia. This same patient also improved from light perception to 20/30 in the CRAO-affected eye.

It is notable that while 54.3 % of treated patients in our study improved by at least 0.3 logMAR, and a few treated patients did experience recovery to baseline or near baseline vision, the majority of patients did not. The mean follow up VA in the treated group was approximately counting fingers. It could be argued that the risk associated with IV thrombolytic might outweigh the benefit, particularly if the fellow eye has normal vision. However, it could also be contended that preservation of vision to any degree possible may be beneficial. A previously published survey of approximately 200 normal-sighted adults reported that, if faced with devastating vision loss in one eye due to CRAO, about 38 % of people would accept treatment-associated risk of additional stroke, and even a small risk of subsequent death, to triple the chances of recovering VA to 20/100 in the affected eye, and more than 80 % of people would accept those risks if the other eye were not sighted.^23^ The importance of well-conducted informed consent to make patients aware of potential risks and benefits of IV thrombolytic treatment for vision loss associated with naCRAO and of the current level of evidence available remains very clear.

Roughly 16 % of fundus photographs were non-diagnostic due to poor image quality. We noted that some of these were uninterpretable poor quality images were taken in emergency rooms soon after camera installation, likely representing the efforts of still inexperienced technicians. The remainder of the non-diagnostic photographs failed to reveal classic CRAO findings such as retinal whitening, a macular cherry red spot, arteriolar boxcarring, or arteriolar plaques. However, the images were still useful to our on-call ophthalmologists as they allowed exclusion of alternative potential causes of acute painless monocular vision loss such as wet macular degeneration, vitreous hemorrhage, or retinal detachment. The use of optical coherence tomography (OCT) at point of contact would likely serve as a useful adjunct to fundus photography to improve the sensitivity of CRAO screening.^24^ In the future, application of deep learning technology to fundus photography may also have great potential to improve early CRAO detection, akin to what has been reported for papilledema.^25^

There are several limitations inherent to this retrospective study. Unstructured documentation and non-standardized measurement of VA, particularly in the ED setting, could lead to inaccurate representation of some presenting VAs. However, this error would presumably apply to both the treated and untreated groups. Follow up VAs were invariably checked in ophthalmology clinics, ensuring a more standardized visual assessment approach. Early Treatment Diabetic Retinopathy Study (ETDRS) testing for these patients may have produced more accurate measurements relative to Snellen acuities.^26^ Visual fields were not consistently performed, and thus could not be analyzed. Length of time to follow up was also not standardized. While we vetted all identified cases coded as CRAO within the study period, lack of correct coding could have resulted in additional cases that were missed. Additionally, CRAO patients with better natural history/spontaneous VA recovery may never present to care or may be counted more often among those patients lost to follow up, potentially skewing conclusions regarding outcomes for untreated patients. Although our study period spanned 2021 to 2023, deployment of fundus cameras and the described treatment pathway was not accomplished until 2022, and this accounts for the smaller number of treated compared to untreated patients in this study. Visual acuity outcomes for the untreated cohort in this study were similar though to previously reported natural history data from earlier cohorts in our patient population.^2^ Finally, while the findings we present highlight the feasibility of implementation in a large-scale real-world setting, they may not be as readily applicable outside of the context of our unique integrated care model. But, our protocol employs telemedicine screening, and we hope that the algorithm presented here could serve as a model for other similar remote diagnostic algorithms.

Directions for future study include: evaluation of the potential role of OCT for detecting cases that are not evident on fundus photography, deep learning for evaluation of fundus photographs, intra-arterial thrombolysis for patients outside of a 4.5 h window, but still presenting within 12 h,^27^^,^^28^ the prospective role of novel neuroprotective agents,^29^ longer term follow up of visual outcomes, evaluation of rates of development of neovascular glaucoma, and the potential for use of IV thrombolysis for patients with branch retinal artery occlusion (BRAO) affecting central vision.

## 5 Conclusion

The evaluation and treatment of hyperacute naCRAO can potentially be achieved on a large-scale via a collaborative, integrated, telemedicine-enabled approach utilizing fundus photography. In our cohort, the use of IV thrombolysis was associated with better VA outcomes compared to observation alone. Ultimately, prospective randomized controlled trials are needed to confirm our findings and determine optimal management.

## CRediT authorship contribution statement

**Aubrey L. Gilbert:** Writing – review & editing, Writing – original draft, Visualization, Validation, Methodology, Investigation, Formal analysis, Data curation, Conceptualization. **Amar P. Patel:** Writing – review & editing, Writing – original draft, Visualization, Validation, Methodology, Investigation, Formal analysis, Data curation, Conceptualization. **Dana Sax:** Writing – review & editing, Writing – original draft, Visualization, Methodology, Investigation, Formal analysis, Data curation, Conceptualization. **M. Tariq Bhatti:** Writing – review & editing, Writing – original draft, Validation, Methodology, Investigation, Formal analysis, Data curation. **Ronak Shah:** Writing – review & editing, Writing – original draft, Visualization, Methodology, Investigation, Formal analysis. **Adrian Dokey:** Writing – review & editing, Writing – original draft, Visualization, Methodology, Investigation, Formal analysis. **Tova Mannis:** Writing – review & editing, Writing – original draft, Visualization, Methodology, Investigation, Formal analysis. **Molly Burnett:** Writing – review & editing, Writing – original draft, Visualization, Methodology, Investigation, Formal analysis. **Robin A. Vora:** Writing – review & editing, Writing – original draft, Visualization, Validation, Supervision, Methodology, Investigation, Formal analysis, Data curation, Conceptualization.

## Patient consent

Written consent to publish this report has not been obtained. This report does not contain any personal identifying information.

## Authorship

All authors attest that they meet the current ICMJE criteria for authorship.

## Funding

No funding or grant support.

## Declaration of competing interest

Aubrey L. Gilbert (No disclosures to report).

Amar P. Patel (No disclosures to report).

Dana Sax (No disclosures to report).

M. Tariq Bhatti (Bristol Meyers Squibb, Consultant).

Ronak Shah (No disclosures to report).

Adrian Dokey (No disclosures to report).

Tova Mannis (No disclosures to report).

Molly Burnett (No disclosures to report).

Robin A. Vora (Iveric Bio, Speaker; Outlook Therapeutics, Consultant, Advisory Board; Paradigm Pharmaceuticals, Consultant; Regeneron, Advisory Board; Genentech, Advisory Board; EyePoint Pharmaceuticals, Consultant).
